# Pharmacological Activities of Mogrol: Potential Phytochemical against Different Diseases

**DOI:** 10.3390/life13020555

**Published:** 2023-02-16

**Authors:** Varun Jaiswal, Hae-Jeung Lee

**Affiliations:** 1 Department of Food and Nutrition, College of BioNano Technology, Gachon University, 1342 Seongnam-daero, Sujeong-gu, Seongnam-si 13120, Republic of Korea; 2 Institute for Aging and Clinical Nutrition Research, Gachon University, Seongnam-si 13120, Republic of Korea; 3 Department of Health Sciences and Technology, GAIHST, Gachon University, Incheon 21999, Republic of Korea

**Keywords:** mogrol, mogroside, phytochemical, pharmacology, anticancer, osteoporosis, antiobesity, anti-inflammation, neurodegenerative disorders

## Abstract

Recently, mogrol has emerged as an important therapeutic candidate with multiple potential pharmacological properties, including neuroprotective, anticancer, anti-inflammatory, antiobesity, antidiabetes, and exerting a protective effect on different organs such as the lungs, bone, brain, and colon. Pharmacokinetic studies also highlighted the potential of mogrol as a therapeutic. Studies were also conducted to design and synthesize the analogs of mogrol to achieve better activities against different diseases. The literature also highlighted the possible molecular mechanism behind pharmacological activities, which suggested the role of several important targets, including AMPK, TNF-α, and NF-κB. These important mogrol targets were verified in different studies, indicating the possible role of mogrol in other associated diseases. Still, the compilation of pharmacological properties, possible molecular mechanisms, and important targets of the mogrol is missing in the literature. The current study not only provides the compilation of information regarding pharmacological activities but also highlights the current gaps and suggests the precise direction for the development of mogrol as a therapeutic against different diseases.

## 1. Introduction

In therapeutics, plant-derived drugs have gained significant importance in recent years, with advancements in the technologies such as analytical techniques, genomics, and engineering strategies [1]. Triterpenes are among the important phytochemicals that are known to have different important biological properties that may be useful as therapeutics [2,3]. Triterpene, mogrol found in the fruits of *Siraitia grosvenorii* recently stretched its importance as a potential therapeutic candidate as it was observed to have different pharmacological activities which can be utilized in the development of therapeutics among different diseases or conditions. These activities of mogrol include neuroprotective, anticancer, anti-inflammatory, antiobesity, antidiabetes, anti-osteoporosis, and anti-colitis activities, and protective activity in lung diseases such as lung injury and pulmonary fibrosis [4,5,6,7]. Mogrol is the aglycone of mogrosides that are the main constituents of the extract of the fruit of *Siraitia grosvenorii* [8]. *S. grosvenorii* is a traditional Chinese medicinal food known for various pharmacological activities, which may be attributed to its several bioactive components, such as mogrol and mogrosides [9]. Some studies have shown that biological activities, such as reduction in triglyceride accumulation and activation of AMPK, were only attributed to mogrol, not to mogroside V, the most abundant mogroside of *Siraitia grosvenorii* [4,6]. Recently, studies have shown that most mogrosides are converted through intestinal digestive enzymes and microflora to mogrol [10,11,12]. Therefore, mogrol may also be the main functional component of mogrosides responsible for pharmacological effects. Recently, considering the high application prospects of mogrol in medicine and the low yield in extraction from plant extract, a de novo biosynthesis pathway was constructed in yeast (*Saccharomyces cerevisiae*) that can be developed as an alternate source for the production of mogrol in place its extraction from the plant [13]. Recently, a multigene stacking strategy is developed to synthesize different mogrosides through metabolic engineering in other plants [14]. Similarly, considering the role of plant-based sweeteners in the environment and economic benefits [15], researchers have created UDP-glycosyltransferase enzymes to synthesize high-intensity sweetener mogrosides from mogrol [16]. Researchers have studied the quantification method of mogrol along with mogroside V in rat plasma after oral and intravenous administration for pharmacokinetic studies [17,18,19]. Along with the different pharmacological activities of mogrol, researchers have also been elucidating the mechanism behind the pharmacological activities to understand and optimize the pharmacological activities of mogrol. In these studies, several important targets such as AMPK, TNF-α, STAT3, P53, and NF-κB were found to be associated with the activity of mogrol, which suggests the role of mogrol may be studied against other important diseases associated with these important targets [20,21,22]. Researchers have also begun to design and test the analogues of mogrol to achieve better pharmacological activity against important diseases such as cancer and targets such as AMPK [23]. However, a comprehensive compilation of the pharmacological activities, associated mechanisms, and targets of mogrol, which could serve as a resource to highlight the current gaps and propose future directions for its development as a therapeutic candidate, is not available.

## 2. Literature Search

A literature search was conducted using online databases, including Scopus, PubMed, Google Scholar, Google, and ResearchGate. In the search, important keywords such as mogrol and its combinations with pharmacology, disease, biological activity, anticancer, antidiabetes, antiobesity, anti-inflammatory, in vivo, and in vitro studies were used. The resulting research articles, review articles, book chapters, and books published in the English language until December 2022 were considered, and the important literature was included in this study.

## 3. Mogrol Structure and Properties

**Figure 1:**
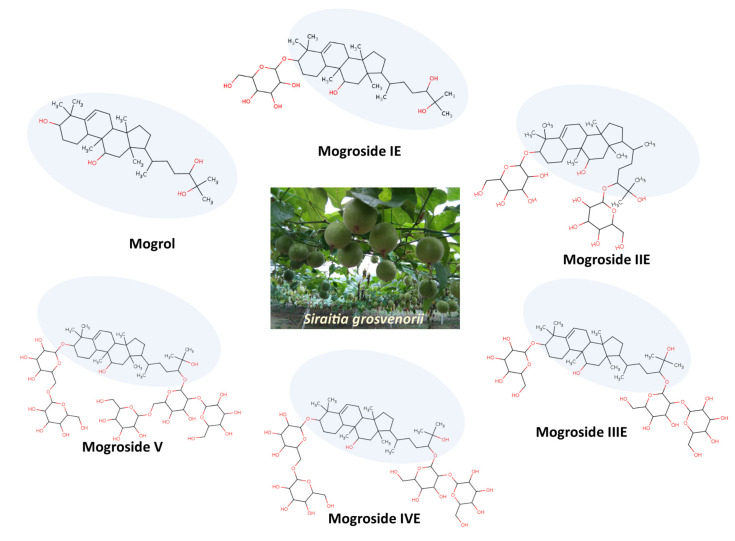
Structure of mogrol and mogrosides found in monk fruit (*S. grosvenorii*). The oval shape highlights the mogrol moiety present in mogrosides.

Mogrol and mogrosides are mainly found in the fruits of *S. grosvenorii* (also known as monk fruit), and the content of these compounds is in the range of 0.55–0.65% in fresh fruit [24]. Mogrol is a cucurbitane-type tetracyclic triterpenoid, and the mogrosides are the group of compounds that contain different glycosylated compounds of mogrol. Mogrol is the precursor of mogrosides in the fruits of *S. grosvenorii*, and mogrol is converted into mogrosides with the growth and ripening of the fruit through the enzyme glycosyltransferases [16]. Several mogrosides were identified from the monk fruit named according to the number of glycosylated sugar moieties which form β-linkages with mogrol. The presence of no sugar moiety in mogrol and more sugar moieties in mogroside V is considered the reason behind the bitterness of mogrol and the high sweetness of mogroside V [16]. The study has shown that the mogrosides on digestion are mostly converted to mogrol. The structure of mogrol and important mogrosides (such as mogrosides IE, IIE, IIIE, IVE, and V) are provided in Figure 1. The molecular weight of mogrol is 476.7 Da, and it contains four hydrogen bond donors and four hydrogen bond acceptors in the structure. Limited toxicology studies have been conducted on mogrol and its synthetic derivatives, but toxicology studies on mogrosides extracted from the fruits are available. In cell proliferation assays, mogrol has no effect on the viability of 3T3-L1, mouse type II alveolar epithelial cell MLE-12, and bone marrow macrophages at the concentration up to 50, 40, and 10 μM, respectively [4,25,26]. Similarly, mogrosides were found to be safe in the study conducted on dogs for 28 and 90 days of oral administration at a dose (3000 mg/kg bw/day). In this study, there was no adverse effect of mogrosides from the monk fruits observed in clinical observations, body weight, food consumption, hematology, blood chemistry, urinalysis, gross necropsy, organ weight, and histopathology [27]. Additionally, in the Ames mutagenicity test, the mogrosides were negative, indicating that they do not have the mutagenicity effect [28]. The pharmacokinetic parameter, such as absolute oral bioavailability and elimination half-life (t_1/2_) of mogrol, were approximately 10.3 ± 2.15% and 2.41 ± 0.11 h, respectively [18].

## 4. Pharmacological Activities of Mogrol

### 4.1. In Vitro Antiobesity and Antidiabetes Activity of Mogrol

The antiobesity and antidiabetes activity of mogrol was studied in different in vitro experiments, and the antiobesity and antidiabetic properties of the crude extract of *S. grosvenorii*, rich in several triterpenes (mainly mogrol) and triterpene glycosides (mogroside V), have been observed in different studies [8,29,30]. To precisely identify the compounds in the extract important for antidiabetic effect, the compounds were derived from the crude extract and studied for the activator activity of adenosine monophosphate-activated protein kinase (AMPK) in the HepG2 cell line [6]. AMPK activation is considered a potential therapeutic strategy for the treatment of metabolic disorders, including diabetes and obesity [31]. Mogrol derived from the extract was among the compounds that can cause the highest increase in AMPK phosphorylation better than the positive control compound (berberine) at all studied concentrations (1, 10, and 20 μM) [6], while the main ingredient of the extract, i.e., mogroside V, had no effect on the phosphorylation of AMPK. The results suggested that the mogrol-induced activation of AMPK has been shown to contribute to the anti-hyperglycemic and antilipidemic properties of the *S. grosvenorii* extract [6].

Later, the effect of mogrol and different mogrosides of *S. grosvenorii* on adipogenesis in 3T3-L1 cells was studied. Mogrol, not mogrosides, was able to suppress the differentiation of 3T3L1 preadipocytes to adipocytes. In the study, only mogrol was found to be effective in reducing the accumulation of triglycerides at both 10 and 20 μM concentrations. The effect of mogrol on AMPK phosphorylation was analyzed with the positive control (acadesine). As in the previous study, mogrol increased AMPK phosphorylation [6]. Similarly, the mRNA level of C/EBPβ was also suppressed by the mogrol treatment observed in the study [4]. 

cAMP response element (CRE)-mediated transcriptional activity was also suppressed by mogrol treatment. Similarly, phosphorylation of cAMP response element-binding protein (CREB) was also suppressed by mogrol. The study concluded that mogrol might be suppressing the lipid accumulation in 3T3L1 adipocytes through activation of AMPK, repression of CREB phosphorylation, and CRE-mediated transcription activity [4]. 

In the same year, the researcher studied the potential of the mogrol- and cucurbitane-type triterpenoid (mogroside V) for the activation of AMPK. Mogrol was found to be the potent AMPK activator that can activate the AMPK heterotrimer, AMPKα2β1γ1, with an EC_50_ of 4.2 μM, which was better than the mogroside V (EC_50_ of 20.4 μM). Further, mogrol and mogroside V were subjected to pharmacokinetics analysis in rats [17]. 

Inspired by their earlier studies of the allosteric activation of AMPK by mogrol, the researcher designed the study to increase the allosteric activation of AMPK through mogrol derivatives at the 24 position. The allosteric activation of AMPK was studied for mogrol and its 21 derivatives. Finally, the most potent compound (derivative with the aliphatic-acyclic group at the 24 position in mogrol) was found to be 20 times more effective than mogrol (EC_50_ of 3.0 ± 0.20 μM) [23]. Overall, the different studies suggest that the antiobesity effect of mogrol may be through reducing CREB phosphorylation, suppressing CRE-mediated transcription, and activating AMPK signaling [4,6,17,23].

### 4.2. In Vitro Anti-Inflammatory Activity of Mogrol 

**Figure 2:**
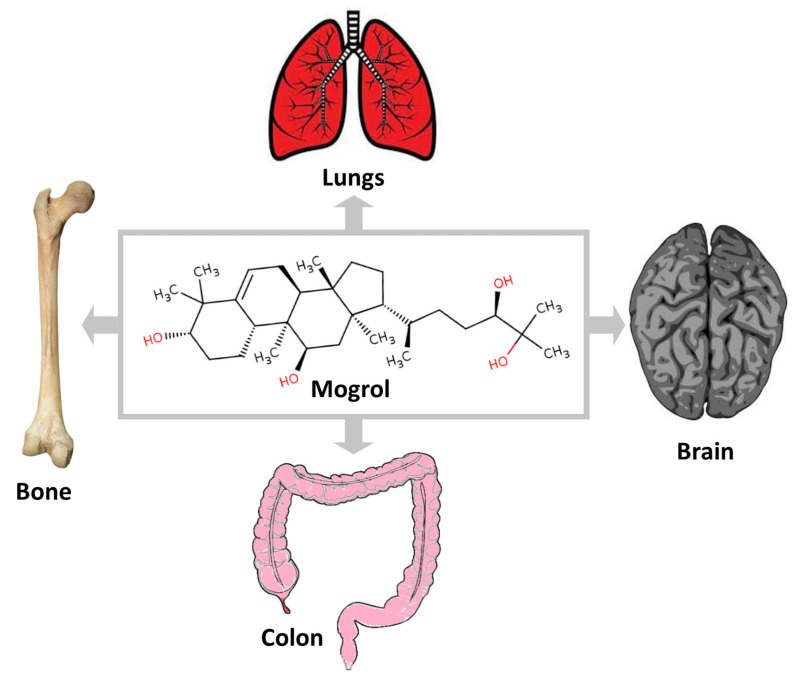
Protective role of mogrol against different organs in animal studies.

The anti-inflammatory activity of mogrol was observed in both in vitro and in vivo studies. Recently, the anti-inflammatory activity of mogrol was evaluated through an in vitro experiment on lipopolysaccharide (LPS)-induced RAW 264.7 cells (Table 1). The application of mogrol at 10 μM concentration significantly reduced the level of pro-inflammatory cytokines (TNF-α, IL-6) and NO production [7]. The anti-inflammatory activity of mogrol was also observed in various in vivo studies in different organs such as the brain, lungs, and colon (Figure 2); these studies are presented in the respective section of the manuscript.

### 4.3. Anticancer Activity of Mogrol 

Initially, the anticancer activities of mogrol have been studied in different cancer cell lines in in vitro experiments [32,33,34]. Later, in vivo studies also supported the anticancer potential of mogrol, and studies were also conducted to decipher the mechanism behind the anticancer activity. Researchers also modified the structure of mogrol to enhance its activity in different studies.

#### 4.3.1. In Vitro Anticancer Activity of Mogrol

In a study, mogrol was derived from *S. grosvenorii* to study its anticancer effect on the human leukemia cell line, K562 cells. The antiproliferative activity of mogrol was studied by the 3-(4,5-dimethylthiazole-2-yl)-2,5-diphenyl tetrazolium bromide (MTT) assay. Mogrol inhibited the growth of K562 cells in a dose- and time-dependent manner (at 0.1, 1, 10, 100, and 250 μM concentration). Further, staining assays reveal the dose-dependent apoptosis of K562 cells through mogrol treatment. Similarly, the population of necrotic/post-apoptotic cells increased significantly (in a dose-dependent manner) to 25.56% from 5.50% with mogrol treatment [32]. Mogrol also caused the growth arrest in the G0/G1 phase of the cell cycle in K562 cells as the percentage of the G0/G1 phase increased from 36.48% to 77.41% with increasing mogrol concentrations. Furthermore, Western blot analysis suggested that mogrol reduces the phosphorylation of ERK1 and ERK2 phosphorylation and also suppresses the level of Bcl-2 in a concentration-dependent manner in K562 cells (Table 1). Similarly, mogrol also suppressed the level of p-STAT3 and improved the expression of the p21 protein in K562 cells, which supports anticancer activity [38,39].

In a study inspired by the earlier reported anticancer activity of mogrol, the anticancer potential of mogrol and synthesized derivatives of mogrol through modification at the C24 and C25 positions was studied in two cancer cell lines (i.e., A549 and CNE1 cell lines). The mogrol derivative resulting from the addition of a tetrahydro-β-carboline structure was found to have the highest antiproliferative activity against both cell lines in the MTT assay [33]. Increased antitumor activity of mogrol derivative (with tetrahydro-β-carboline) may be the intramolecular synergistic effect as the tetrahydro-β-carboline compounds reported for antitumor activity in the literature [40].

Similarly, the anticancer activity of mogrol and a series of novel synthesized derivatives of mogrol was further studied on human lung cancer cells such as A549 and NCI-H460. The in vitro cytotoxicity of the mogrol and synthesized mogrol derivatives were studied through the CCK8 assay against both human lung cancer cell lines. Like the previous studies, the mogrol has shown a significant antiproliferative effect on the A549 cell line (IC_50_ of 27.78 ± 0.98 μM) [33]. Some derivatives of mogrol, especially the replacement of hydroxyl groups at C11 with ester containing the 1,2,3-triazole ring, exhibited significant antiproliferative activity against both cell lines [34]. Like earlier studies on mogrol, the mogrol derivative was also shown to significantly increase the proportions of cells in G0/G1 and down-regulate the level of p-STAT3 in a dose-dependent manner [32]. 

Recently, mogrol and synthesized three series of novel mogrol derivatives were used for anticancer activity in non-small cell lung cancer cell lines. Similarly, mogrol treatment significantly reduced the cell proliferation of lung cancer cell lines (A549 and H1975) [7]. In the study, mogrol, such as indole fused derivatives and derivatives containing α, β-unsaturated ketone moiety, had better anti-inflammatory and anticancer activities than mogrol, respectively [7].

Further, considering the insight from the anticancer activity of the synthesized derivative of mogrol in previous and preliminary studies, the C11 and C3 locations in mogrol were selected for the synthesis of two series of ester derivatives of mogrol. The activity of mogrol and its derivatives was studied in different human cancer cell lines (A549, NCI-H460, and CNE1). In comparison with mogrol, most of the derivatives (especially the esterification in the C11 position of mogrol) were found to have higher antiproliferative activity against cancer cell lines. Like mogrol in the previous study, the effective derivatives of mogrol also suppressed the STAT3 expression in the study, which suggests a similar action mechanism as mogrol. The study supports that the inhibition of STAT3 may be an important mechanism for anticancer activity and is expected to help the development of mogrol-based anticancer drugs [35]. 

Recently, the anticancer effect of mogrol on four cancer cell lines of human lung cancer (A549, H1299, H1975, and SK-MES-1) with normal control bronchial epithelial cells (HBE) was studied before animal experiments. A significant decrease in the survival rate was observed in all cell lines A549, H1299, H1975, and SK-MES-1, in a concentration-dependent manner. Additionally, the surface area (morphology) and the number of lung cancer cells A549, H1299, and SK-MES-1 decreased significantly with the mogrol treatment. Mogrol also inhibits the migration of all studied cell line lung cancer cells but has no significant effect on non-cancerous HBE in the scratch-wound migration assay. The autophagy marker LC3 was found to be significantly increased with mogrol treatment in all four cancer cell lines as compared to respective controls. Further, the adenovirus expressing fluorescent-mRFP-GFP-LC3 was used to visualize autophagic flux; the mogrol significantly increased the autophagosome (yellow dots) as well as autolysosome (green dots) numbers in all four lung cancer cells. 

Further, in both A549 and SK-MES-1 cells, the mogrol treatment significantly increased the ratios of p-AMPK/AMPK in a concentration-dependent manner, along with the increased LC3-II level and decreased cell survival rate. Supplementation with compound c (AMPK inhibitor) significantly reversed the level of LC3II and decreased the autophagosomes. Furthermore, mogrol treatment also significantly increased p53 phosphorylation compared to the respective control in A549 and SK-MES-1 cells. Furthermore, the protein expression of cell cycle-dependent proteins such as p53, PUMA, and cell cycle inhibitory proteins p21 and p27 increased significantly with the mogrol treatment. Additionally, mogrol treatment in both A549 and SK-MES-1 cells resulted in a significant decrease in the levels of antiapoptotic protein (Bcl-2) and the ratios of Bcl-2 to Bax. Encouraging results of mogrol in all four lung cancer cells inspired further animal study [36]. 

#### 4.3.2. In Vivo Anticancer Activity of Mogrol

In the in vivo experiment, male thymus-deficient mice (BALB/C, 20 g) were used for anticancer activity. Lung cancer cells (A549) were injected subcutaneously into the mice, and mogrol treatment started when the tumor size increased to 5 × 5 mm^3^. The weight and volume of the tumor decreased (by 69.18% and 66.22%, respectively) significantly in the mogrol treatment group compared to the control mice group. Additionally, two weeks of mogrol treatment did not show a significant difference in the body weight of tumor-bearing mice with and without mogrol treatment. Similarly, there was no significant effect on the parameters of the cardiac function of mice in echocardiography between mogrol treatment and control mice [36].

### 4.4. Mogrol Activity against Ulcerative Colitis

Earlier reported anti-inflammatory activity of mogrol encouraged the analysis of the mogrol against ulcerative colitis (UC), a chronic inflammatory bowel disease [37]. Initially, the significant activity of mogrol against UC was observed in the mice model, and the mechanism behind the anti-colitis activity was explored in in vitro cell line experiments.

#### 4.4.1. In Vivo Anti-UC Activity of Mogrol

The dextran sulfate sodium (DSS)-induced mice were used as the model for UC in the study. Mogrol, at a dose of 5 mg/kg body weight (BW), weakly prevented weight loss and colon shortening, and reduced the disease activity index (DAI) caused by DSS in the mice (Table 2). Inhibitive effects of mogrol on the inflammatory infiltration in colonic tissues were also observed in the histopathological examination. Additionally, the mogrol significantly decreased the level of mRNA of IL-17 (anti-inflammatory factor) and increased the level of IL-10 (pro-inflammatory factor). The activation of NLRP3 inflammasome was markedly repressed in the mogrol treatment group, which is known to be activated in UC. Similarly, the levels of mRNA of NLRP3 and IL-1β were also reduced in the colons of mice of mogrol treated group. Furthermore, the mogrol also distinctly inhibited the degradation of IκBα. These results support that mogrol can suppress too great a release of inflammatory factors and also suppress the activation of inflammation-related pathways to support the prevention of colon damage in a similar way to how the Mesalazine drug protects against DSS-induced damage in UC [37]. The study supports the protective role of mogrol through its inflammatory effect, which may be effective for other organs (Figure 2). 

Mogrol also protected the integrity of tight junction (TJ) in the intestinal epithelium, as mRNA and protein expression of tight junction proteins (occludin and ZO-1), which were down-regulated due to DSS application in the colon tissues of mice, significantly increased with mogrol treatment. Similarly, mogrol treatment also significantly raised the mRNA and protein expression of SIRT1as compared with DSS administered group. AMPK has been known to enhance intestinal barrier function and can restore the assembly of TJ. In previous studies, the mogrol is known as an AMPK activator; hence, AMPK phosphorylation was also studied [4,6]. The mogrol treatment significantly promoted AMPK activation, which was reduced due to DSS-induced colitis in mice [37].

#### 4.4.2. In Vitro Anti-Colitis Activity of Mogrol

Furthermore, to study the mechanism behind the mogrol intestinal epithelial cell homeostasis with mogrol in colitis, TNF-α-stimulated NCM460 cells (human intestinal epithelial cells) were utilized.

Mogrol treatment at (10 μM) significantly restored the protein expression of occluding and ZO-1, which was significantly reduced by the TNF-α, and there was no effect on the proliferation of normal cells. Additionally, the mogrol treatment reorganized the flat-shaped microtubule network of tubulin stimulated by TNF-α, which may stimulate the assembly of the tight junction (Table 1). Similarly, Mogrol treatment elevated the bcl-2/bax ratio, which was significantly reduced with the application of TNF-α; these results support the role of mogrol in the homeostasis of intestinal epithelial in inflammatory conditions (Table 1). Further, mogrol is the AMPK activator found to increase the phosphorylation of AMPK in the TNF-α-stimulated NCM460 cells, and this may be the main reason for the protective activity of mogrol, as coadministration of mogrol with compound C (an AMPK inhibitor) results in a reversal of the protective activity, such as a reduction in both AMPK phosphorylation and the expression of TJ proteins (ZO-1 and occludin) [37].

Similarly, macrophages (THPM) derived from the THP-1 cells, stimulated with LPS and ATP, were utilized for mogrol treatment to study its role in inflammatory cytokines. Similarly, like NCM460 cells, without affecting normal cell proliferation, the mogrol treatment at 10 μM significantly reduces the caspase-1 activation and the secretions of IL-1β. The effect of mogrol in the study appeared to be similar to that of metformin (a positive drug) [37].

### 4.5. Activity of Mogrol against the Pulmonary Fibrosis 

The activity of mogrol was also studied in different experiments (in vitro and in vivo) against pulmonary fibrosis (PF), a chronic interstitial lung disease that does not have an effective treatment [43]. Furthermore, an increase in air pollution and occupational exposure to metal or wood dust may worsen the risk of PF [44].

#### 4.5.1. In Vitro Anti-PF Activity of Mogrol

The epithelial–mesenchymal transition (EMT) is one of the key mechanisms concomitants with PF associated with the down-regulation of E-cadherin and up-regulation of α-SMA, type Col I, and Vimentin. In the study, Mogrol was found to reverse the expression of E-cadherin, α-SMA, type Col I, and Vimentin in TGF-β1-treated mouse type II alveolar epithelial cells MLE-12 at both 5 and 10 μM concentrations. Similarly, in primary lung fibroblasts (PLFs) cells, the up-regulated protein levels of α-SMA, Lysyl oxidase-like protein 2 (LOXL2), collagen I, and phosphorylation forms of Smad2/3 due to TGF-β1 were restored with the mogrol treatment at 10 μM concentration (Table 1). AMPK activation and NOX4 activity can stimulate lung myofibroblast differentiation, and mogrol also significantly restored the unusually decreased p-AMPK and amplified NOX4 protein expression in TGF-β1-treated PLFs [26]. 

#### 4.5.2. In Vivo Anti-PF Activity of Mogrol

Furthermore, to study the antifibrotic role of mogrol in animals, a bleomycin-induced lung fibrosis model (Male C57BL/6 mice) was used. In the bleomycin-induced PF mice group, an increase in pulmonary index and a decrease in weight and survival rate were observed. Mogrol treatment significantly reduced weight loss and pulmonary index compared to the bleomycin group at both doses (5 or 10 mg/kg), similar to the antifibrotic effects of the nintedanib (a positive drug at a dose of 40 mg/kg) group [26,45]. The histology of the lungs in the staining study revealed bleomycin-induced alveolar wall thickening, neutrophil infiltration, edema, and higher collagen production in the lung tissues compared to normal. In the mogrol treatment group, these lung damages were distinctly inhibited by decreasing the area of collagen fibrils and inflammatory degree compared to the bleomycin group. Like in the in vitro study, in the mogrol treatment group, the protein and mRNA expression of α-SMA and Col I, which was increased in the bleomycin group, was decreased in lung tissue. Similarly, the expression of LOXL2 in lung tissues was significantly decreased in the mogrol (high dose) and nintedanib groups. Further, to investigate the mechanism behind the antifibrotic mechanism of mogrol through the TGF-β1/Smad2/3 signaling pathway, the expression of mRNA and proteins of TGF-β1 and Smad2/3 phosphorylation was reduced in the mogrol treatment group; their expression was enhanced in the bleomycin-administered group. Importantly, like in vitro experiments, compared to the bleomycin group, the mogrol treatment group also had a significant increase in the activation of AMPK in lung tissues. Mogrol also significantly restored abnormal activation of bleomycin-induced deacetylase sirtuin 1 (Sirt1) compared with the bleomycin group in lung tissue. Sirt1 is important in TGF-β1 signaling and myofibroblast transdifferentiation; hence, both TGF-β1/Smad and the AMPK/Sirt1 signaling pathways may be important for mogrol activity. Moreover, nuclear receptor subfamily 4 group A 1 (NR4A1), which was increased in the bleomycin group, is known to be important in both fibrogenesis and TGF-β1 signaling and was significantly suppressed in the mogrol treatment group. Similarly, mogrol also suppressed bleomycin-induced NOX4 mRNA and protein expression in the lung tissue. Interestingly, coadministration of compound c (AMPK inhibitor) with mogrol reversed the protective effect of mogrol, i.e., the bleomycin-induced lung inflammation and fibrosis process in mice. AMPK activation may be the important mechanism for mogrol activity; hence, molecular docking analysis was also conducted to study the interaction and binding energy of mogrol with AMPK, and mogrol had a slightly better docking score than AMP. Results from the study suggest the anti-fibrosis activity of mogrol results at least partly from the activation of AMPK [26,46]. 

### 4.6. Anti-Osteoporosis Activity of Mogrol 

Osteoporosis is a common disease around the globe, and elders and especially elderly women, are in the high-risk group [47]. The antiosteoporosis activity of mogrol was also discovered in both in vitro and in vivo studies. 

#### 4.6.1. In Vitro Antiosteoporosis Activity of Mogrol

The bone marrow macrophages (BMM) were stimulated with a receptor activator of nuclear factor-kB ligand (RANKL) treated with different concentrations of mogrol. The tartrate-resistant acid phosphatase (TRAP) positive multinucleated osteoclasts were decreased with the treatment of mogrol in a dose-dependent manner (at 5, 10, and 20 μM). The early stages (1–3 days) of osteoclast formation were especially strongly inhibited by the mogrol. The mogrol also shows an inhibitory effect on osteoclast resorption function. The bone resorption in the bovine bone slices implanted with BMM after the formation of osteoclast cells was studied. Scanning electron microscopy examination has shown that the mogrol significantly reduced the resorption area in a dose-dependent manner.

RNA-seq analysis can be used to decipher the gene expression mechanism associated with the treatment [48,49]. To study the gene expression changes behind the mogrol activity on RANKL-induced BMMs, RNA-seq analysis was used, which showed that the osteoclastogenesis suppressive genes (lilrb4a and Fcgr3) were up-regulated and osteoclastogenesis genes (MMP9, OSCAR, and ACP5) were down-regulated with the mogrol treatment. Further, the reduced expression of marker genes of osteoclastogenesis (such as MMP9, CTSK, ATP6v0d2, ACP5, and DCSTAMP) in the mogrol treatment was confirmed through RT-PCR analysis (Table 1).

Mogrol was found to suppress the MAPK/NF-κB signaling pathway by suppressing the phosphorylation of JNK, ERK, P65, and p38. Mogrol also suppressed the degradation of IκBα and nuclear translocation of P65, which is important for the NF-κB activity supporting the antiosteoporosis activity [50].

The expression of downstream transcription factors c-FOS and NFATc1, which was significantly increased in BMM activated with RANKL, was found to be reduced through mogrol treatment [25].

#### 4.6.2. In Vivo Antiosteoporosis Activity of Mogrol

Encouraging osteoporosis activity of mogrol in in vitro experiments paves the path for in vivo studies. In vivo osteoporosis activity of mogrol was conducted through a mouse model after ovariectomy (OVR). After 42 days of mogrol treatment (at the dose of 10 mg/kg), Micro-CT 3D reconstructions showed decreased bone loss of the femurs in OVR mice. Quantitative results showed that the bone volume/tissue volume (BV/TV), trabecular thickness (Tb. Th), trabecular number (Tb. N), and cross-sectional thickness (Cs. th) of the mogrol treatment group were distinctly greater than the vehicle group. Further, in histological assessment, the BV/TV was again found to be higher in the mogrol group (Table 2). The immunohistochemistry TRAP staining of femurs showed a reduction in osteoclast surface/bone surface area (Oc. S/BS) and the number of osteoclasts/bone perimeter (N. Oc/B) of the mogrol treatment group compared to the vehicle group [25]. Both in vitro and in vivo studies suggested the role of mogrol in the suppression of osteoporosis, and it may be developed as a therapeutic against osteoclast-mediated osteolytic disorders such as osteoporosis (Table 2) [25].

### 4.7. In Vivo Neuroprotective Activity of Mogrol

The anti-inflammatory activity of mogrol was the motivation behind the basis to analyze its neuroprotective activity against neurotoxic conditions due to inflammatory agents such as LPS and amyloid β-peptide (AB) [51,52].

First, the neuroprotective activity of mogrol was studied in mouse models by analyzing memory impairment caused by AB. In both the Morris water maze test and Y-maze test, mogrol has significantly reduced AB-induced memory impairment in ICR mice at all doses used in the study (20, 40, 80 mg/kg). Mogrol also reduced the over-activation of microglia, as Iba1-positive cells in the hippocampus, which were significantly increased due to AB application, were reduced with mogrol treatment. Similarly, mogrol also inhibited the Hoechst-positive cells of the dentate gyrus (DG). The mechanism behind the neuroprotective activity of mogrol may be the reduction in NF-κB mediated neuroinflammation, as AB can induce the expression of NF-κB and associated inflammatory factors. In further experiments, mogrol treatment was found to suppress the NF-κB p65 and associated inflammatory factors such as IL-1β, IL-6, and TNF-α (Table 2) [41]. Similarly, the mogrol inhibited the ratio of cleaved-caspase-3 and pro-caspase-3 found to be increased due to AB, and increased the ratio of Bcl-2 and Bax found to be decreased due to AB toxicity.

**Figure 3:**
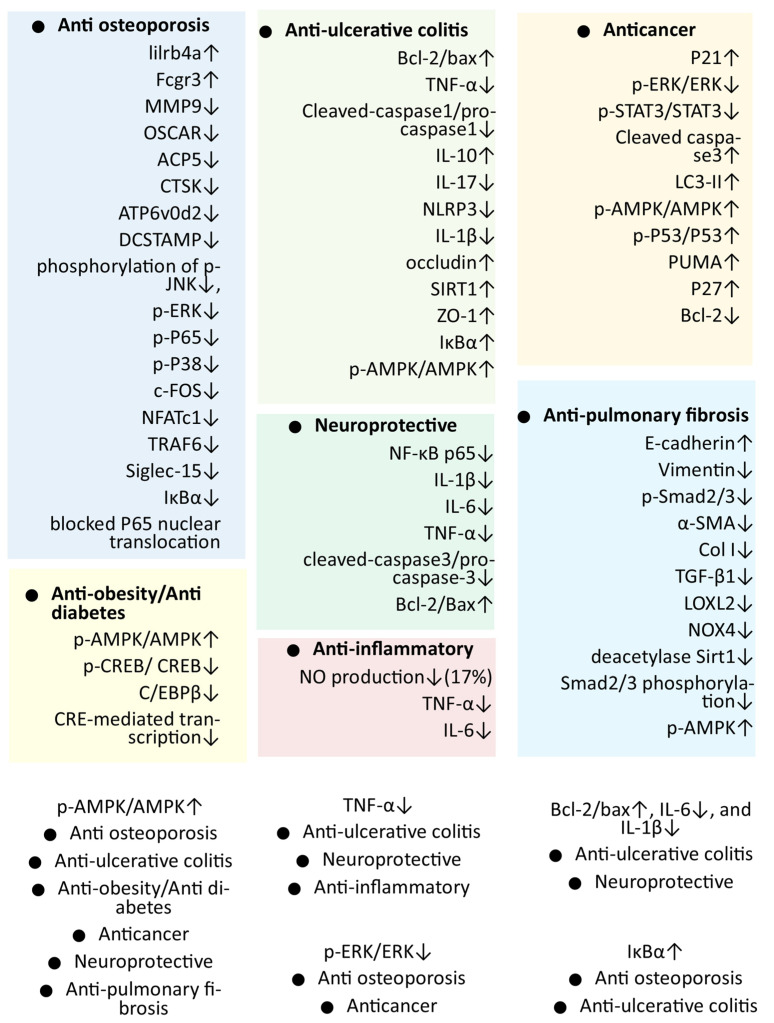
Different targets/markers were found to be associated with the pharmacological activities of mogrol. (Studied genes common in more than one activity are listed with white background and upward (↑) and downward (↓) arrows represent significantly up-regulated and down-regulated genes respectively).

Later, in a similar study, the neuroprotective effect of mogrol against lipopolysaccharide (LPS)-induced memory impairment and neuroinflammatory responses was studied in mice. In memory impairment tests such as the Morris water maze, Y-maze, and novel object recognition tests, mogrol treatment significantly reduced the memory impairment in ICR mice caused due to LPS at all doses used in the study (20, 40, 80 mg/kg). Like in the previous study, Mogrol reduced microglial overactivation as it reduced the Iba1-positive cells in the hippocampus that increased significantly increased due to LPS. Similarly, mogrol was found to suppress the NF-κB p65 and associated inflammatory factors such as IL-1β, IL-6, and TNF-α in both the frontal cortex and hippocampus region of the brain (Figure 3) [42]. In both studies, it can also be concluded that the protective effect of mogrol in the brain may be at least partly associated with the anti-inflammatory activity of mogrol (Figure 2).

## 5. Discussion and Future Directions

The positive result of recent studies evaluating the pharmacological activity of mogrol accelerates the efforts for the development of mogrol as a therapeutic agent. The protective effect of mogrol on different organs also supported its therapeutic potential against different diseases (Figure 2). Pharmacokinetic studies also support the potential of mogrol as a drug candidate compared to mogrosides. Still, the development of mogrol as a therapeutic or supplement required considerable effort in the precise direction. The antiobesity and antidiabetic activity of mogrol, which are mainly based on the activation of AMPK, were studied in various studies. However, AMPK is the crucial target of metabolism/energy-related diseases. However, there is a need for in vivo experiments that could establish antiobesity and antidiabetic activities before clinical evaluation. Similarly, the anticancer effect of mogrol was observed in different cancer cell lines, suggesting its anticancer effect against important cancers, including lung cancer, but limited animal studies have been conducted for its support. Hence, more animal studies are required and suggested for the anticancer activity of mogrol before clinical studies [53]. The anti-inflammatory activity of mogrol is also one of the most promising activities, which might be the important factor behind its other pharmacological properties, such as neuroprotective, anticancer, and anti-UC activities [37,41,42,54,55]. Along with important inflammatory mediators such as (IL-6 and TNF-α), it was found that it can suppress NF-κB, which is an important central component in the pathways involved in various important diseases related to chronic inflammation such as different cancer, rheumatoid arthritis, inflammatory bowel diseases, neuro-inflammatory-related diseases, etc. [41,42,56,57]. It would be interesting to see the effect of mogrol on these diseases. Therefore, it is suggested to study the role of mogrol against chronic inflammation-related diseases.

The neuroprotective activity of mogrol observed in both studies was protective against memory impairment in a mouse model by suppressing inflammatory pathways, especially NF-κB signaling. Neuroprotection studies may be further required to establish with more experiments, mainly through different animal models, before clinical evaluation. Similarly, mogrol may also be studied against other neurodegenerative diseases and disorders in which neuro-inflammation is an important component of disease etiology, such as Parkinson’s disease [58].

However, the anti-PF activity of mogrol was studied only through two cell-based experiments and one animal experiment. It could be a valuable activity to consider in future studies as PF is a deadly disease with no cure [43]. In other studies, the lung protective effect of mogrol was also observed [4]. However, the development of mogrol as a therapeutic against PF is in the preliminary stage, and more animal models are proposed to be used in the near future to develop mogrol as a therapeutic against it. Importantly, the role of AMPK activation was observed as one of the important molecular mechanisms for PF activity. Hence, the activity of mogrol derivatives that were found to be better AMPK activators may also have been suggested for their anti-PF activity.

A similar suggestion can be followed for anti-UC studies in the near future, such as the PF study on AMPK inhibitors that can reverse the positive effect of mogrol on anti-UC activity. An important aspect is the molecular mechanism behind pharmacological activities, which was also significantly explored for various activities of mogrol (Figure 3).

The molecular mechanism gives an insight into the possible therapeutic targets that may be used to further develop and optimize the effect of therapeutic [59]. Finally, it can be summarized that the activation of AMPK is an important mechanism for most of the pharmacological activities of mogrol, including antiobesity, antidiabetes, anticancer, anti-ulcerative colitis, anti-PF, and antiosteoporosis. Further, anti-inflammation through suppression of the NF-κB pathway and/or inflammatory cytokines is an important mechanism that also supports different pharmacological activities of mogrol, including neuroprotective, anti-UC, anti-PF, and anticancer activities. Inhibition of ERK and STAT3 and activation of the p53 pathway were also found to be important contributors to the anticancer activity of mogrol. Similarly, the CREB activation may also contribute to the antiobesity and diabetes activity of mogrol. Pathways and/or genes found to be targeted in different experiments and associated with more pharmacological activities of mogrol can be considered as important reliable targets to be developed mogrol and its derivatives as therapeutic.

Derivatives of mogrol are synthesized by modifying the chemical group in the selected position in the mogrol. Some of these derivatives have been found to have increased pharmacological activities compared to mogrol, such as anticancer and AMPK activator [7,23]. The binding of mogrol through in silico molecular docking also provided the initial understanding of the interaction of mogrol with its important target AMPK [26]. It is suggested to use similar docking studies to rationally design and synthesize the mogrol derivative for increased activity with AMPK and, subsequently, better biological activities. Overall, mogrol is a potential phytochemical that can be taken for further evaluation in the in vivo pharmacological experiments consulting the currently proposed suggestion, and later clinical studies may be conducted after safety evaluation.

## Figures and Tables

**Table 1:** Pharmacological activities of mogrol studied in the in vitro experiments.

Activity (and Probable Mechanism)	Dose	Method	Result	Ref.
Antiobesity/antidiabetes (through reducing CREB activation and promoting AMPK activation)	1, 10, and 20 μM	HepG2 cell line AMPK activator (phosphorylation of AMPK)	p-AMPK/AMPK ↑ more efficiently than berberine	[6]
	1, 5, 10, and 20 μM	3T3-L1 cells Oil red O staining and classical Folch method	At 20 μM lipid accumulation ↓ and cellular TG levels ↓	[4]
	20 μM	WB	p-AMPK/AMPK ↑ and p-CREB/CREB ↓	
	20 μM	Real-time PCR	C/EBPβ ↓	
	20 μM	Reporter assay	CRE-mediated transcription ↓	
	0.625–40 μM	AMPK activations through HTRF assay	A769662 (EC_50_ of 24.9 nM) and AMP (EC_50_ of 1.4 nM) EC_50_ of 4.2 μM.	[17]
	mogrol	HepG2 cells AMPK allosteric activator screening based on SPA assay	EC_50_ = 3.0 ± 0.20 for mogrol and 1.4 ± 0.10 μM for AMP	[23]
Anti-inflammatory (inhibition of TNF-α mediated inflammation)	10 μM of mogrol	RAW 264.7 cells NO production	NO production ↓ (17%)	[7]
	10 μM of mogrol	ELISA	The levels of TNF-α ↓ and IL-6 ↓	
Anticancer (autophagy and autophagic cell death via activating AMPK signaling pathway, ERK, and STAT3 inhibition)	0.1, 1, 10, 100, and 250 μM	Cell proliferation (K562 cells) study through MTT assay	Growth inhibition 88% at 250 μM.	[32]
		WB	P21 ↑, Bcl2 ↓, p-ERK/ERK ↓, and p-STAT3/STAT3 ↓	
	50 and 100 μM	The A549 and CNE1 cell lines MTT assay	IC_50_ (μM) 89.51 ± 3.95 (A549) 81.48 ± 4.73 (CNE1)	[33]
	30 μM	CCK8 against lung cancer cell lines (A549 and NCI-H460)	IC_50_ (μM) 27.78 ± 0.98 (A549) >100 (NCI-H460)	[34]
		A549, NCI-H460, H1299 and H1975	IC_50_ (μM) 28.56 ± 1.98 (A549) >100 (NCI-H460) >100 (H1299) 87.14 ± 2.56 (H1975)	[7]
		A549, NCI-H460, and CNE1 CCK8 assay	IC_50_ (μM) 27.78 ± 0.98 (A549) >100 (NCI-H460) >100 (CNE1)	[35]
	50 μM	A549, H1299, H1975, and SK-MES-1 analyzed through CCK-8 kit and celigo cell counter	IC_50_ (μM) <25 μM	[36]
	50 μM	Inverted confocal microscopy and surface area were measured by ImageJ	Surface area ↓ and the number of lung cancer ↓ in A549, H1299, and SK-MES-1 cells	
	50 μM	Scratch-wound migration assay	Migration of all studied cells ↓	
	50 μM	WB	LC3-II ↑	
	50 μM	Adenovirus expressing fluorescent-mRFP-GFP-LC3	Autophagosomes ↑, as well as autolysosome ↑	
	50 μM	A549 and SK-MES-1 cells WB	Cleaved caspase3 ↑, LC3-II ↑, p-AMPK/AMPK ↑, p-P53/P53 ↑, PUMA ↑, p21 ↑, p27 ↑, and Bcl-2 ↓	
Anti-colitis (through promoting AMPK activation and inhibition of TNF-α mediated inflammation)	pre-treated with mogrol (1 or 10 μM)	NCM460 human intestinal epithelial cells stimulated through TNF-α (metformin (the positive control drug), 2 mM used as control in the study)	Occludin ↑, ZO-1 ↑, bcl-2/bax ↑, TNF-α ↓, and p-AMPK/AMPK ↑ at (10 μM of mogrol)	[37]
	10 μM	THP-1 cells were stimulated by PMA to macrophages and stimulated by LPS and ATP (metformin as positive control drug used in the study)	Cleaved-caspase1/pro-caspase1 ↓, and IL-1β ↓	
Antifibrotic activity (through activation of AMPK-mediated signaling pathways and inhibition of NF-κB signaling pathways)	1, 5, and 10 μM of mogrol	TGF-β1-treated mouse type II alveolar epithelial cells (MLE-12 cell line) Expression analysis through WB	E-cadherin ↑, α-SMA ↓, type Col I ↓, and Vimentin ↓	[26]
	1, 5, and 10 μM of mogrol	PLFs cells expression analysis through WB	α-SMA ↓, (LOXL2 ↓), collagen I ↓, and phosphorylation forms of Smad2/3 ↓ and increased p-AMPK ↑	
	10 μM of mogrol	The protein expression was measured by immunofluorescence staining	NOX4 ↓ protein expression in TGF-β1-treated PLFs	
Anti-osteoporosis (inhibition of TRAF6/MAPK/NF-κB signaling pathway)	(0, 5, 10, 20 μM)	BMM stimulated with RANKL	Osteoclasts ↓ (236.67 ± 37.07 to 20.0 ± 6.08) at 20 μM mogrol.	[25]
	0, 5, 10, 20 μM	Bone resorption in bovine bone slices through SEM	Bone resorption ↓	
	20 μM	RNA-Seq	lilrb4a ↑ and Fcgr3 ↑ osteoclastogenesis suppressive genes) MMP9 ↓, OSCAR ↓, and ACP5 ↓ (osteoclastogenesis genes)	
	20 μM	RT–PCR was utilized to confirm osteoclastogenesis marker genes expression	MMP9 ↓, CTSK ↓, ATP6v0d2 ↓, ACP5 ↓, and DCSTAMP ↓	
	20 μM	WB	Phosphorylation of JNK ↓, ERK ↓, P65 ↓, and P38 ↓ Expression of c-FOS ↓, NFATc1 ↓, TRAF6 ↓, and Siglec-15 ↓ Suppressed the degradation of IκBα ↓	
	20 μM	Immunofluorescence staining	Mogrol efficiently blocked P65 nuclear translocation, which is required for NF-κB activation	

AICAR: 5-aminoimidazole-4-carboxamide-1-beta-D-ribofuranoside; AMPK: adenosine monophosphate-activated protein kinase; CCK8: Cell Counting Kit-8 assay; DSS: dextran sulfate sodium; ELISA: enzyme-linked immunosorbent assay; HTRF: homogeneous time-resolved fluorescence assay; LOXL2: lysyl oxidase-like protein 2; LPS: lipopolysaccharide; MTT: 3-(4,5-dimethylthiazole-2-yl)-2,5-diphenyl tetrazolium bromide; PLFs: primary lung fibroblasts; PMA: phorbol myristate acetate; Sirt1: sirtuin 1; SPA: scintillation proximity assay; WB: Western blotting analysis; ↑: significantly up-regulated; ↓: significantly down-regulated.

**Table 2:** Pharmacological activities of mogrol studied in the in vivo experiments.

Activity (and Probable Mechanism)	Model	Dose	Method	Results	Ref.
Anticancer (activating AMPK signaling and p53 pathway)	Male thymus-deficient mice (BALB/C)	10 mg/Kg three times a week for 2 weeks	Mogrol injected intraperitoneally into the mice	The weight and volume of tumor decreased (by 69.18% and 66.22%, respectively	[36]
Anti-UC (through promoting AMPK activation and inhibition of inflammation)	Female C57BL/6 mice DSS-induced mouse UC model	Mogrol (1 or 5 mg/kg) for 7 days orally	Weight monitoring, DAI, and colon length calculation (metformin as positive control drug used in the study)	Weight loss ↓, DAI ↓, and colon shortening ↓	[37]
			Histopathological examination (metformin as positive control drug used in the study)	Inflammatory infiltration in colonic tissues ↓	
			RT-PCR	IL-10 ↑, IL-17 ↓, NLRP3 ↓, IL-1β ↓, occludin ↑, SIRT1 ↑, and ZO-1 ↑	
			WB	IκBα ↑, occludin ↑, ZO-1 ↑, and p-AMPK/AMPK ↑	
Anti-PF (through activation of AMPK-mediated signaling pathways and inhibition of NF-κB signaling pathways)	Male C57BL/6 mice	1, 5, and 10 mg/kg	The lung was used for measuring pulmonary index	Positive drug nintedanib Pulmonary index ↓ and weight loss ↓	[26]
		1, 5, and 10 mg/kg	Histology of lungs (Nintedanib used as positive drug group)	Alveolar wall thickening ↓, neutrophil infiltration ↓, edema ↓, high collagen production ↓, area of collagen fibrils ↓ and inflammatory degree ↓	
		1, 5, and 10 mg/kg	Expression analysis of lung tissues through RT-PCR and WB	α-SMA ↓, Col I ↓, and TGF-β1 ↓	
		10 μM of mogrol	Expression analysis in lung tissues through WB	LOXL2 ↓, NOX4 ↓, deacetylase Sirt1 ↓, Smad2/3 phosphorylation ↓, and p-AMPK ↑ in lung tissues	
Antiosteoporosis (inhibition of TRAF6/MAPK/NF-κB signaling pathway)	Female C5BL/6J mice ovaries were removed	10 mg/kg mogrol intraperitoneally every second day for 42 days	Micro-CT 3D reconstructions indicated that mogrol decreased bone loss of femurs in OVX mice	BV/TV ↑, Tb. Th ↑, Tb. N ↑ and Cs. Th ↑ of the mogrol group were considerably higher than the vehicle group; BS/BV ↓ was inversely lower	[25]
			Histological assessment	BV/TV ↑ of the mogrol group was dramatically higher than that of the vehicle group	
			Immunohistochemistry TRAP staining of femurs	Oc. S/BS ↓ and N. Oc/B ↓ of the mogrol group were substantially lower than those of the vehicle group	
Neuroprotective (suppression of NF-κB mediated inflammation)	Male ICR mice AB-induced memory impairment	20, 40, 80 mg/kg intragastric administration	Morris water maze test and Y-maze test (2 mg/kg donepezil as positive control drug used in the study)	At all doses, significantly improved memory impairment	[41]
			Immunohistochemical analyses (donepezil as positive control drug used in the study)	Iba1-positive cells↓	
			Hoechst assay	Number of Hoechst-positive cells of the DG ↓	
			WB	NF-κB p65 ↓ IL-1β ↓, IL-6 ↓, TNF-α ↓, cleaved-caspase3/pro-caspase-3 ↓ and the Bcl-2/Bax ↑	
	Male ICR mice LPS-induced memory impairment	20, 40, 80 mg/kg intragastric administration	Morris water maze test, Y-maze test, and novel object recognition test	In all the tests, mogrol improved memory impairment	[42]
			Immunohistochemical analyses	Iba1-positive cells ↓	
			WB of mouse in the mouse hippocampus and frontal cortex	NF-κB p65 ↓ IL-1β ↓, IL-6 ↓, and TNF-α ↓	

AB: amyloid β-peptide; AICAR: 5-aminoimidazole-4-carboxamide-1-beta-D-ribofuranoside; AMPK: adenosine monophosphate-activated protein kinase; BS/BV: bone surface/bone volume; BV/TV: bone volume/tissue volume; CCK8: Cell Counting Kit-8 assay; Cs. Th: cross-sectional thickness; DAI: disease activity index; DG: dentate gyrus; DSS: dextran sulphate sodium; HTRF: homogeneous time-resolved fluorescence assay; LOXL2: lysyl oxidase-like protein 2; LPS; lipopolysaccharide; MTT: 3-(4,5-dimethylthiazole-2-yl)-2,5-diphenyl tetrazolium bromide; N. Oc/B: number of osteoclasts/bone perimeter; Oc. S/BS: osteoclast surface/bone surface area; PF: pulmonary fibrosis; PLFs: primary lung fibroblasts; PMA: phorbol myristate acetate; SEM: scanning electron microscopy; Sirt1: sirtuin 1; SPA: scintillation proximity assay; Tb. N: trabecular number; Tb. Th: trabecular thickness; UC: ulcerative colitis; WB: Western blotting analysis; ↑: significantly up-regulated; ↓: significantly down-regulated.

## Data Availability

Not applicable.
